# Predicting the affective tone of everyday dreams: A prospective study of state and trait variables

**DOI:** 10.1038/s41598-019-50859-w

**Published:** 2019-10-14

**Authors:** Eugénie Samson-Daoust, Sarah-Hélène Julien, Dominic Beaulieu-Prévost, Antonio Zadra

**Affiliations:** 1 Department of Psychology, Université de Montréal, Montreal, QC Canada; 2 Department of Sexology, Université du Québec à Montréal (UQAM), Montreal, QC Canada; 3 Center for Advanced Research in Sleep Medicine (CARSM), Hôpital du Sacré-Coeur de Montréal, Montreal, QC Canada

**Keywords:** Human behaviour, Physiology, Human behaviour, Physiology

## Abstract

Although emotions are reported in a large majority of dreams, little is known about the factors that account for night-to-night and person-to-person variations in people’s experience of dream affect. We investigated the relationship between waking trait and state variables and dream affect by testing multilevel models intended to predict the affective valence of people’s everyday dreams. Participants from the general population completed measures of personality and trauma history followed by a three-week daily journal in which they noted dream recall, valence of dreamed emotions and level of perceived stress for the day as well as prior to sleep onset. Within-subject effects accounted for most of the explained variance in the reported valence of dream affect. Trait anxiety was the only variable that significantly predicted dream emotional valence at the between-subjects level. In addition to highlighting the need for more fine-grained measures in this area of research, our results point to methodological limitations and biases associated with retrospective estimates of general dream affect and bring into focus state variables that may best explain observed within-subject variance in emotions experienced in everyday dreams.

## Introduction

Despite decades of advances in dream research, relatively little is known about how dreams are formed and what factors predict their content and emotional tone. One of the most widely studied models of dream content is the continuity hypothesis of dreaming^1,2^ which posits that dreams are generally continuous with the dreamer’s current thoughts, concerns and salient experiences. In line with this conceptualization of dreams, a large proportion of dream research^1,3–7^ has been dedicated to quantifying various dimensions of people’s dream reports and investigating their relationship to different aspects of people’s waking life. While much of this work has helped refine our understanding of which aspects of waking life (e.g., day-to-day actions, ongoing concerns, learning tasks, stressful experiences, psychological well-being) are most likely to be reflected or embodied in various facets of people’s dreams (e.g., settings, interpersonal interactions, activities, thematic contents), attempts to identify factors accounting for night-to-night or person-to-person variations in the intensity and valence of dream affect have yielded mixed results^7–13^.

Given that emotions are present in a vast majority of home and laboratory dream reports^7,14–17^ and that some theorists^18–20^ believe that affect plays a key role in structuring dream content, elucidating why people experience negatively toned dreams on some nights and positively toned dreams on others is of prime importance. Among the most studied factors hypothesised to influence dream valence are stress^21–24^, trait or personality characteristics^25–27^, history of traumatic experiences^28–31^, and psychological well-being^7,18,32,33^. Relatedly, one neurocognitive model^34,35^ of dysphoric and everyday dream production suggests that variations in the frequency and intensity of negative dream emotions are partially determined by *affect load*, or day-to-day variations in emotional stress, and that the relation between dream content and stress varies as a function of *affect distress*, or the disposition to experience events with distressing, reactive emotions.

Many of the factors believed to predict the experience of negative dreams, including trauma history and psychopathology, have been associated with disturbed dreaming^28,36–38^ and likely contribute to the development and heightening of affect distress^34,39^. Similarly, other dispositional traits related to the concept of affect distress, such as boundary thinness^40^ (used to describe particularly sensitive and vulnerable individuals prone to mixing thoughts, images and feelings) and trait anxiety^41^ (stable individual differences in the tendency to experience anxiety across situations) are also correlated with indices of negative dream content, including frequency of bad dreams and nightmares^27,33,42–45^. Thus, affect distress may be viewed as encompassing a range of factors known to impact dream affect, including trauma history, psychopathology, trait anxiety, and boundary thinness.

While several studies have investigated the differential impact of state and trait factors on dream content^7,11,12,32,42,46–50^, most have focused solely on nightmares, have been purely retrospective in nature, or did not weigh state-related findings against trait factors such as personality or psychopathology. Only two studies^42,48^ have ever used a prospective design to assess the effect of trait and daily state measures on everyday dreams. The first one^42^ assessed state anxiety and depression (what the authors termed “mood”) in relation to trait measures believed to underlie nightmare occurrence. They found statistically significant correlations between their state and trait variables and nightmare frequency, but only in individuals with thin psychological boundaries. The second study^48^ obtained similar results in that daily stress was found to statistically predict general sleep-related experiences—a concept elaborated by Watson^51^ to describe nocturnal phenomena such as nightmares, falling dreams, flying dreams and sleep paralysis—but only in young adults scoring high on a measure of trait dissociation (the tendency to experience psychological detachment from reality).

In sum, in addition to giving rise to inconsistent results, research on the determinants of dream affect has been limited by the often retrospective nature of the study design, single measurement points, focus on nightmare incidence or broad sleep-related experiences, and a failure to evaluate the interactive role of state and trait factors within a larger conceptual framework. We therefore used a prospective, multilevel design to investigate the interplay between daily fluctuations in perceived levels of stress and trait indices of affect distress as determinants of dream affect. Individuals from the general population first completed questionnaire measures of sleep and dream experiences, trait anxiety, boundary thinness, trauma history, and PTSD symptoms, followed by at least three consecutive weeks of daily assessments of perceived stress as well as dream recall, including the emotional valence associated with each remembered dream. Since daily measures (*N* = 2538) were nested within individuals (*N* = 128), multilevel hierarchical linear modelling (HLM) analyses were performed in order to examine the distinctive effect of state and trait variables.

## Results

### Descriptive statistics and intercorrelations of tested variables

Table 1 presents the means, standard deviations and zero-order Pearson correlations between study variables. Daily measures were averaged per participant over the study’s duration to investigate their association to trait variables. All observed correlations were in the expected direction. The highest obtained correlation (*r* = 0.752) was between the mean daily level of maximum stress and the mean level of stress prior to bedtime. The fact that daily maximum stress was more strongly correlated with daily dream valence (*r* = 0.300) than was daily stress prior to bedtime (*r* = 0.185) suggests that the two variables tapped into different facets of perceived stress. As can be seen in the table, trait anxiety was statistically correlated with a majority of other studied variables, while sex did not show statistically significant correlations with any of the other measures.

**Table 1:** Correlations, Means and Standard Deviations of Trait and State Study Variables (*N* = 128).

	*M*	*SD*	*Trait variables (Level-2)*						*State variables (Level-1)*		
			Sex	Age	STAI-T	BQ18	ETISR-SF	PC-PTSD	M-Stress	B-Stress	DRF
Sex											
Age	42.55	14.63	−0.017								
STAI-T	44.11	10.46	−0.033	−0.164							
BQ18	33.14	9.12	0.056	−0.123	0.336***						
ETISR-SF	2.18	1.94	−0.080	−0.106	0.115	0.146					
PC-PTSD	1.04	1.34	0.053	−0.006	0.373***	0.273**	0.342***				
M-Stress	3.73	1.60	0.012	−0.169	0.332***	0.162	0.046	0.184*			
B-Stress	1.83	1.45	−0.040	0.023	0.361***	0.114	0.019	0.242**	0.752***		
DRF	1.58	0.74	0.140	0.203*	−0.192*	0.076	−0.045	−0.107	−0.151	−0.142	
Dream valence	5.12	1.10	−0.023	−0.103	0.288***	0.105	0.047	0.097	0.300***	0.185*	−0.052

*Note*. Pearson correlations (two-tailed). Correlations with sex represent point-biserial correlations. Women were coded 0, men were coded 1. Correlations with daily measures are for mean value or total value (for DRF) per participant across study duration, while their means and standard deviations are across all measurement occasions (i.e., 2653 days). *STAI-T* = trait scale of the State-Trait Anxiety Inventory - Form Y; *BQ18* = short form of the Boundary Questionnaire; *ETISR-SF* = shortened French version of the Early Trauma Inventory-Self Report; *PC-PTSD* = Primary Care PTSD screen; *M-Stress* = maximum perceived stress; *B-Stress* = bedtime perceived stress; *DRF* = dream recall frequency. *p < 0.05. **p < 0.01. ***p < 0.001.

### Multilevel models predicting dream valence as outcome

**Figure 1:**
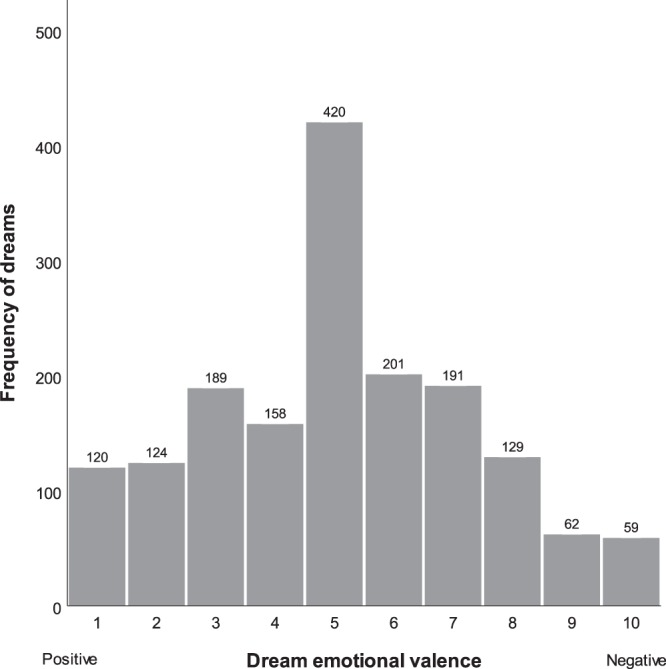
Distribution of dream emotional valence for 1653 dream reports.

A total of 1700 nights led to a dream recall in participants over the study’s three-week duration, of which 1653 (97.2%) contained ratings on the dream’s emotional valence. Of the 1700 nights, 773 (45.5%) yielded more than one recalled dream and participants reported an average of 6.9 dreams per week. Figure 1 presents the distribution of dream valence ratings for the 1653 dream reports. The mean dream valence score was 5.08 (*SD* = 2.27), or at the midpoint of the positive to negative rating scale. As can also be seen in the figure, highly positive dreams (scores of 1 or 2) were approximately twice as frequent as highly negative ones (scores of 9 or 10).

Table 2 presents the intercepts-only model (i.e., unconditional model) for daily measures of dream valence. The intraclass correlation was 0.161, indicating that 16.1% of the variance in dream valence occurred between subjects, while 83.9% of the variance occurred within subjects (i.e., across days).

**Table 2:** Covariance Parameters of Intercepts-Only Model for Predicting Dream Valence.

	*Level*	*Estimate*	*SE*	*Wald Z*	*p*	95% CI	
						*LL*	*UL*
Within-subject variance	1	4.301	0.155	27.713	<0.001***	4.007	4.616
Between-subject variance	2	0.825	0.148	5.584	<0.001***	0.581	1.172

*Note. SE* = standard error. Based on 1653 observations from 128 participants. *p < 0.05. **p < 0.01. ***p < 0.001.

Table 3 presents the multilevel model predicting dream valence using trait (Level-2) and state (Level-1) predictors. At Level-2, when all predictors were entered in the model as fixed terms, trait anxiety (STAI-T) was the only variable to statistically predict dream valence. At Level-1, neither of the two daily measures of perceived stress statistically predicted the dream valence experienced on the subsequent night. Dream recall frequency per night was the only statistically significant Level-1 predictor. This measure was used as a control variable since dream valence was only provided for the best remembered dream on a given night when more than one dream was recalled (45.5% had multiple recalls) and thus the two variables were not entirely independent.

**Table 3:** Linear Mixed-Effects Model for Dream Valence.

	*Estimate*	*SE*	*df*	*t*	*p*	95% CI	
						*LL*	*UL*
Intercept	4.743	0.219	248.605	21.668	<0.001***	4.312	5.174
*Between-subject predictors (Level-2)*							
Sex	−0.078	0.235	102.552	−0.332	0.741	−0.544	0.388
Age	−0.003	0.007	118.613	−0.405	0.686	−0.017	0.011
**Trait anxiety (STAI-T)**	**0.032**	**0.011**	**111.621**	**2.909**	0.004**	**0.010**	**0.053**
Boundary thinness (BQ18)	−0.006	0.012	112.021	−0.475	0.636	−0.030	0.018
Youth trauma (ETISR-SF)	−0.017	0.057	108.177	−0.293	0.770	−0.130	0.096
PTSD symptoms (PC-PTSD)	0.018	0.085	104.872	0.214	0.831	−0.150	0.186
*Within-subject predictors (Level-1)*							
Maximum stress	0.012	0.031	1328.583	0.378	0.706	−0.049	0.073
Bedtime stress	−0.006	0.047	1281.578	−0.126	0.899	−0.098	0.086
*Control variable (Level-1)*							
Dream recall frequency (DRF)	0.278	0.083	1382.530	3.345	<0.001***	0.115	0.441

*Note*. Trait anxiety and boundary thinness scores were grand mean centered (Level-2), while maximum and bedtime stress were participant mean centered (Level-1). *SE* = standard error, *df* = degrees of freedom. Based on 1653 observations from 128 participants. *p < 0.05. **p < 0.01. ***p < 0.001.

When standardized scores for trait anxiety (ZSTAI-T) were entered as a single predictor of dream valence in a separate model, it was found to be an even better predictor (*p* < 0.001) than when it was considered alongside other predictor variables, with each increase in standard deviation STAI-T scores explaining a 0.33 unit increase in dream valence ratings. This model reduced the unexplained between-subject variance by 11.6%, thus explaining a total of 1.9% of the variance in dream valence ratings obtained over the study’s 3-week duration.

### Post Hoc multilevel models predicting dream valence as an outcome variable

Since interactions between predictors could potentially explain why neither of our perceived stress variables predicted dream valence^42,48^, we tested for possible interactions, particularly between trait variables (Level-2) and daily perceived stress (Level-1), but did not find a statistically significant interaction that could predict dream valence. The only statistically significant interaction predicting dream valence was between trait anxiety (STAI-T) scores and dream recall frequency (*p* = 0.007), which was positive and expected since the dream valence rating of the most vivid or best-remembered dream on a given night can increase when a greater number of dreams is recalled on that night.

Since daily perceived stress did not predict the dream valence experienced on the subsequent night, models testing for potential a dream-lag effect (i.e., increased incorporation in dreams of events having occurred 5–7 days prior to the dream)^52,53^ were also computed post hoc. Separate datasets pairing daily perceived stress levels from previous days (i.e., two to seven days prior to recalled dreams) with reported valence of subsequently recalled dream were generated. No statistically significant effect of perceived stress from the past 2 to 7 days on dream valence was found in any of the datasets tested, thus refuting a possible delayed effect of perceived stress on subsequently experienced dream affect.

### Additional multilevel models predicting perceived stress as outcome

Using a reversed model, we aimed to predict daily stress scores (both maximum and prior to bedtime) using dream valence and DRF from the preceding night, along with the other predictor variables. The models only yielded a statistically significant effect of trait anxiety as a predictor of both maximum (*p* = 0.031) and bedtime stress levels (*p* = 0.007) (see Supplementary Tables S1 and S2 for more details).

## Discussion

We investigated the relationship between waking trait and state variables and dream affect by testing multilevel models aiming to predict the affective valence of people’s everyday dreams. Moreover, this was the first time a prospective day-by-day design was used to test predictors of dream valence at the between-subject as well as within-subject levels of variance. The results showed that daily measures of perceived stress collected from a non-clinical sample of adults do not, as suggested by some theorists, predict the emotional valence of dreams experienced later that night, nor on immediately subsequent nights. This study is also the first to identify trait anxiety as a key dispositional variable in predicting dream valence, even when trait measures are weighed against state variables.

Taken as a whole, these results run counter to previous findings indicating that state variables are better predictors of dysphoric dream frequency than are dispositional traits^46,47^, and that daily stress or mood interacts with trait variables to predict nightmares^42,48^. Previous positive results could be due to methodological considerations as these studies either lacked a multilevel, prospective design, focused on nightmare occurrence^42,46,47^ or general sleep-related experiences^48^ instead of everyday dreams, or focused on undergraduate (often psychology) students instead of recruiting participants from the general adult population^46–48^.

Our results are reminiscent of Cellucci and Lawrence’s study^49^ of nightmare sufferers showing that daily ratings of general and maximum anxiety were statistically correlated with nightmare frequency and intensity in only a small minority of participants. Since trait variables were not assessed in their study, why nightmare occurrence was related to daily anxiety in some participants but not others remains to be determined. In line with this question, Soffer-Dudek and Shahar^48^ found that daily stress predicted “general sleep-related experiences” only in individuals scoring high in trait dissociation (a trait strongly correlated with boundary thinness), while Blagrove and Fisher^42^ found that correlations between state anxiety and nightly incidence of nightmares were only statistically significant in participants scoring high on boundary thinness. While the interplay between dispositional and state factors underlying nightmare occurrence may play a role in the emotional tone of everyday dreams, the current study showed no statistical interactions between various trait variables and daily levels of perceived stress in predicting dream valence.

With respect to the other dispositional traits investigated, it is noteworthy that although traumatic experiences, including aversive events during one’s childhood, are well-documented correlates of disturbed dreaming^21,34,54–56^, we found no statistically significant effect of trauma history on everyday dream affect. Most findings linking trauma and dream content, however, have come from work focused on trauma-related nightmares, typically in patients diagnosed with PTSD. By contrast, only 23 (18%) of our participants had a cut-off score of 3 or greater on the PC-PTSD (indicative of ongoing trauma-related difficulties) and only 16% reported more than one dream with an affect score of 9 or 10 (indicative of a nightmare) during the three weeks of the study. In fact, as shown in Fig. 1, dreams with highly intense negative affect represented less than 8% of the over 1600 dream reports collected in the current study.

Similarly, while boundary thinness has been linked to dream content variables such as high dream recall, frequent nightmares and negatively-toned dreams^26,43,57–59^, it had no predictive value in our models of everyday dream valence. This trait variable may be better suited to the study of nightmare sufferers, a population specifically investigated by Hartmann *et al*.^59^ when developing this personality construct, or to individuals prone to particularly vivid or bizarre dreams^26^.

Turning to the construct of affect load, the current study did not find evidence to support the idea that daily variations in perceived stress are temporally related night-to-night variations in dream affect. It should be noted that studies having reported an effect of affect load on the emotional content of dreams did so by measuring affect load retrospectively (e.g., for the past month) at a single point in time^7,46,47^ rather than on a day-to-day basis. This underscores the importance of how state factors are assessed since correlates of retrospectively estimated state variables can be biased by dispositional factors (e.g., personality) and are not necessary correlates of prospective, day-to-day measurements of these constructs. In fact, this is not the first time in dream research that prospective study designs have yielded findings contradicting results obtained with retrospective measurements of dream-related variables, including correlates of dream recall and dream content^60–62^.

The concept of affect load may also need to be better defined to allow for more directly comparable study results. For example, in exploring the effects of stress on dreams, researchers have investigated acute stressors^63,64^, experimental stressors^22,65^, emotional stressors^66^, as well as cumulative stressors^21^. Additionally, in light of the recently proposed social simulation theory of dream function^67^ in which dreaming is conceptualized as simulating social skills and bonds to strengthen waking social relationships, the study of social or interpersonal stressors^68^ in relation to dream content may be particularly valuable, especially since a vast majority of dream reports feature social interactions^5,15,69^ and that concerns of an interpersonal nature are frequent in everyday dreams^1,3^. Moreover, as suggested by some researchers^50^, dream content may be more reactive to the emotional nature of stressors than to the stressors *per se*. Finally, it is important to note that our participants were not particularly stressed—or at least did not perceive that they were—during the 3-week study as reflected by their mean score of 3.6 (out of 9) on our measure of daily maximum stress and 1.7 (out of 9) for daily bedtime stress. It is possible that direct or interaction effects of state and trait variables on dream affect become heightened, and thus more readily observable, during periods of acute or chronic stress.

When stress or affect load are studied in relation to dream content, they are usually assessed with self-report questionnaires. However, subjective levels of perceived stress can differ from variations or patterns in the biological markers of cortisol^70,71^. It is thus possible that physiological modulation of stress response, as opposed to subjective stress perception, plays a role in people’s nightly experience of dream affect. Of note, Nagy *et al*.^72^ found a blunted cortisol awakening response in women reporting frequent nightmares, which was independent of lifestyle, psychiatric symptoms and demographic variables. This led the authors to hypothesize that low cortisol reactivity could be a trait-like feature of nightmare sufferers. Similarly, some researchers^73^ have suggested that the gradual rise in people’s cortisol level from the middle of the night until its peak in the morning could account for observed increases in dream emotionality, bizarreness, vividness and length across the night^74^, independently of sleep stage. The use of biomarkers such as cortisol, which can be sampled in saliva^72^, could therefore be of particular interest in investigating the range and intensity of dream emotions reported both within and across nights.

Furthermore, since dream emotional valence was measured for the best-recalled dream upon awakening in the morning, the current study is limited to a narrow portion of participants’ sleep mentation. In addition, given the recency of morning dreams^75^ and the aforementioned increase in dreamlike qualities of sleep mentation across the night, dream emotional valence was likely based on dreams occurring moments before morning awakenings. Affect load could thus have been processed through the emotional valence of dreams that were not collected in the present study (i.e., dreams from earlier periods of the night or other forms of unrecalled sleep mentation). Such a hypothesis could be tested with serial laboratory-based awakenings for dream collection across the sleep period, although the proportion of dreams containing emotions as well as their valence tend to differ when they are self-reported in the laboratory^13,14,17,76^ as opposed to participants’ natural home enviornment^16,77–79^.

Finally, our sample of over 1600 dream reports revealed a roughly equal distribution of positive and negative emotions, as well as a higher proportion of intense positive emotions as opposed to negative emotions. This finding adds to the growing evidence showing that when the presence and valence of dreamed emptions are scored by the participants themselves as opposed to by external judges, as done in early studies of dream content^15^, a considerably higher proportion (70% to 100%) of dream reports are found to contain emotions^16,77–79^ and that positive dream affect is particularly more frequent than when dream reports are assessed by external raters^17,79^. These findings also highlight the interest of investigating positive dimensions of waking states, such as mindfulness^27^ and positive emotions^7^ in relation to dream affect. In a related vein, the study of how self-regulation techniques such as relaxation and meditation may modulate the impact of state and trait factors on dream content also merits investigation.

In sum, results of the present study showed that trait anxiety, but not day-to-day levels of perceived stress, predicted the affective tone of home dream reports and revealed a potential bias in previous studies associated with the use of one-time retrospective assessments of state variables in predicting night-to-night variations in dream affect. The present results also underscore the need for additional research on factors underlying the valence of emotions experienced in everyday dreams as opposed to focusing solely on nightmares or trauma-related dreams. In particular, the study of different categories of stressors and the use of stress biomarkers could be particularly useful in elucidating the differential impact of state and trait factors on dream content.

## Method

### Procedure

Data were collected as part of a larger online study conducted on the Qualtrics Research Suite platform. After providing informed consent, participants were emailed a link giving them access to the study materials. Participants first completed a series of questionnaires on sleep, personality, trait anxiety and trauma history. They then received, over a maximum of four consecutive weeks, daily scheduled notifications to complete a questionnaire on dream recall in the morning as well as an evening questionnaire on the stress and emotions experienced that day. The project was approved by the Arts and Science Research Ethics Committee of the Université de Montréal, Canada (Project no. CERAS-2017-18-013-P) and all research was performed in accordance with their guidelines and regulations.

### Participants

One hundred and twenty-eight non-paid participants (98 women, 30 men, *M*_*age*_ = 42.55, *SD*_*age*_ = 14.63, range = 19–76 years) were recruited from the general adult population between February and July 2018 via ads in free local newspapers (74.9% of sample), social networks (9.4%), email lists (8.6%) and community posters (7.1%). Study materials were available in both French and English to reflect the bilingual nature of Montreal, Canada. One hundred and twelve of the 128 volunteers (87.5%) completed the study in French. Eighty-eight participants (68.8% of sample) were working at the time of study, 20 (15.6%) were students, 12 (9.4%) were retired, 5 (3.9%) were unemployed, and 3 (2.3%) did not specify their occupation. Of the 285 people who initially expressed interest in the study, 151 provided written informed consent and completed the first set of questionnaires. Of these 151 participants, 23 (18 women, 5 men) were excluded for providing fewer than three consecutive days of matching stress and dream valence data. Participants’ morning dream data were paired with their stress ratings completed prior to bedtime the night before. Sixty-six of 128 participants (51.6%) completed one or more days of data collection beyond the 21 consecutive days required. These data were included in the analyses as they contained validly paired evening stress and morning dream valence scores.

### Retrospective measures

Participants first completed a general Sleep and Dream Questionnaire^33^ used to assess basic sleep, dream and demographic variables.

#### Boundary thinness

The short form of the Boundary Questionnaire (BQ18)^80^, which contains 18 items derived from the original Boundary Questionnaire^40^, was used to measure boundary thinness or thickness, a personality trait associated with various aspects of dreaming^57^, including high dream recall^43^ and nightmare prevalence^58^. People with thin psychological boundaries are typically described as being creative, sensitive, vulnerable and easily mixing thoughts, images and feelings. The total score of the BQ18 consists of a sum of the ratings (ranging from 0 to 4) on the 18 items after inverting the ratings on 4 items. Scores on the BQ18 are positively correlated (*r* = 0.87, *N* = 856) with total scores on the original Boundary Questionnaire^80^. Cronbach’s alpha (α) for the BQ18 in the present study was 0.70.

#### Trait anxiety

The Trait scale of the State-Trait Anxiety Inventory – Form Y (STAI-T)^81^ measures anxiety as an enduring personality trait and consists of 20 statements that pertain to how participants “generally feel.” Each item is rated on a 4-point Likert scale. The total score is calculated as a sum of all the ratings (ranging from 0 to 80), with a higher score indicating higher trait anxiety. The STAI-T is widely used and has been translated in multiple languages, including in French Canadian^82^. The latter shows a correlation of *r* = 0.82 with the original English version and a test-retest correlation of *r* = 0.94. The original French-Canadian translation shows strong internal consistency (α = 0.91) and an identical reliability (α = 0.91) obtained in the present study.

#### Youth trauma

A shortened French version^83^ of the Early Trauma Inventory Self Report (ETISR-SF)^84^ was used to assess a range of physical, emotional, and sexual abuse experiences that may have occurred before the age of 18. The seven items, presented in “Yes-No” format, yield a total score ranging between 0 and 7. Cronbach’s alpha (α) for the ETISR-SF in the present study was 0.73.

#### Posttraumatic stress disorder

The Primary Care PTSD Screen (PC-PTSD)^85^ measures four factors specific to posttraumatic stress disorder (PTSD): reexperiencing, avoidance, hyperarousal and numbing. A positive response to any of the yes/no items indicates that the responder may have PTSD or trauma-related problems, and a cut-off score of 3 is recommended to detect positive cases. Cronbach’s alpha (α) for the PC-PTSD in the present study was 0.75.

### Prospective measures

Dream recall and content were assessed each morning via URL links emailed to each participant at 3:00 AM. To ensure that reported dream recall data was for the targeted day, daily links expired at 6:00 PM. This time range was sufficiently broad to accommodate participants’ occupations and schedules. Reminders were automatically sent out at 3:00 PM if the morning questionnaire had not been completed by that time. Waking perceived stress for the day was measured prior to bedtime with links sent out at 6:00 PM and expiring at 3:00 AM. A reminder was sent at 12:00 AM (i.e. midnight) if participants had not completed the evening questionnaire by that time.

#### Dream affect and content

Dream recall was assessed with a single item, “Did you dream last night?” and a “Yes-No” answer format. If “No” was selected, participants had the option of returning to the questionnaire if ever they remembered a dream later in the day. If participants answered “Yes,” they were required to indicate if they remembered one, two, or three or more dreams from that night. These values were used to calculate participants’ dream recall frequency. Participants then had to indicate (for the most vivid or best-remembered dream from the night if more than one dream was recalled), the dream’s emotional valence by answering the question, “What was the general emotion of your dream?” using a 10-point Likert scale ranging from positive (1) to negative (10).

#### Perceived stress

Two daily measures of perceived stress were completed prior to bedtime using a 10-point Likert scale ranging from not stressed at all (0) to extremely stressed (9). The first measure required participants to rate the maximum level of stress experienced that day while the second required participants to rate their stress level at the time of questionnaire completion (i.e., prior to bedtime). These scales, reviewed by Dr. Sonia J. Lupien, director of the Centre for Studies on Human Stress (https://humanstress.ca/), were used instead of more exhaustive instruments such as the Daily Stress Inventory^86^ due to the multi-week nature of the study and our desire to limit volunteers’ workload.

### Statistical analyses

Data were analyzed using hierarchical linear modeling (HLM) with IBM SPSS Statistics (version 25), where affect load (level 1: affective dream content [outcome], perceived stress [predictor]) was underpinned by the participants’ dispositional measures (level 2 predictors: trait anxiety, boundary thinness, trauma history, PTSD, sex, age). The level of statistical significance for every analysis was set at *p* = 0.05. This type of multilevel analysis is ideally suited to such a dataset as it a) allows for the analysis of multiple relationships while considering shared variance at both levels, b) takes into account dependency across measurement time points, c) doesn’t require balanced designs in which different individuals have a fixed number of prospective data points without any missing data, and d) has fewer assumptions and is less likely to underestimate error than other statistical methods^87^.

Although dream valence was the main outcome variable of interest, models predicting daily perceived stress were also tested to investigate possible effects of dreamed emotions on daytime stress. Dream valence had a normal distribution and enough anchor points (10) to approximate continuity. It was thus tested using linear mixed-effects modeling (MIXED command). Since both measures of daily perceived stress were positively skewed, they were tested under a Poisson distribution using a generalized estimating equation (GENLIN command) which, in both cases, presented a better model fit than with a normal distribution under a linear mixed-effects model.

When dream valence was the outcome variable, measures of daily stress from the preceding day were used as Level-1 predictors while trait, trauma and demographic variables were used as Level-2 predictors. Since dream recall frequency was measured daily, it was also used as a Level-1 predictor to assess its possible mediating effect on dream valence and other predictor variables, with values from 1 (one dream remembered on that night) to 3 (three or more dreams remembered). When daily stress was the outcome of interest, the dataset was shifted in order for a given night’s dream valence to be paired with levels of perceived stress of the following day. Considering that participants’ first daily measurement was for perceived stress, there was a smaller total of 2410 observations, not 2538, because the first stress values and last dream valence values were unpaired and thus excluded.

We first computed an intercepts-only model where time was not specified as a repeated measures variable and no predictors entered. This procedure is recommended to determine the amount of between-subject variance in the outcome variable, also known as the intraclass correlation^88^. The intraclass correlation was thus calculated by dividing the value of the intercept (between-group) variance by the sum of the residual (within-group) variance and intercept.

We then progressively added predictors to the unconditional model, beginning with individual Level-2 predictors. All Level-2 variables were grand mean centered. Level-1 stress predictor variables were centered to each participants’ mean for the duration of the study to account for dispositional biases in reported self-ratings.

Finally, post hoc analyses were performed to test alternate hypotheses. Interactions were tested between predictors to assess whether the model generalized to the whole sample or if some effects were moderated by other variables. We individually tested and reported the potential moderating effects of every level 2 predictor and of dream recall and valence (level 1) on each of the two level 1 stress predictors. The effect on dream valence of the stress variables from 2 to 7 days ago was also tested using lagged independent variables.

## Supplementary information

Supplementary Tables

## Data Availability

The datasets generated during and/or analysed during the current study are available from the corresponding author on reasonable request.

## References

[1] Domhoff G. William (1996). Finding Meaning in Dreams.

[2] Hall, C. S. & Nordby, V. J. *The individual and his dreams*. (Signet, 1972).

[3] Domhoff, G. W. *The emergence of dreaming: mind-wandering, embodied simulation, and the default network*. (Oxford University Press, 2018).

[4] Han HJ, Schweickert R, Xi Z, Viau-Quesnel C (2016). The cognitive social network in dreams: transitivity, assortativity, and giant component proportion are monotonic. Cogn. Sci..

[5] Pesant N, Zadra A (2006). Dream content and psychological well-being: a longitudinal study of the continuity hypothesis. J. Clin. Psychol..

[6] Schredl M, Erlacher D (2008). Relation between waking sport activities, reading, and dream content in sport students and psychology students. J. Psychol..

[7] Sikka P, Pesonen H, Revonsuo A (2018). Peace of mind and anxiety in the waking state are related to the affective content of dreams. Sci. Rep..

[8] Davidson J, Lee-Archer S, Sanders G (2005). Dream imagery and emotion. Dreaming.

[9] Gilchrist S, Davidson J, Shakespeare-Finch J (2007). Dream emotions, waking emotions, personality characteristics and well-being — a positive psychology approach. Dreaming.

[10] Nixon A, Robidoux R, Dale AL, De Koninck J (2017). Pre-sleep and post-sleep mood as a complementary evaluation of emotionally impactful dreams. Int. J. Dream Res..

[11] Schredl M (2006). Factors affecting the continuity between waking and dreaming: emotional intensity and emotional tone of the waking-life event. Sleep Hypn..

[12] Schredl M, Reinhard I (2010). The continuity between waking mood and dream emotions: direct and second-order effects. Imagin. Cogn. Pers..

[13] St-Onge M, Lortie-Lussier M, Mercier P, Grenier J, De Koninck J (2005). Emotions in the diary and REM dreams of young and late adulthood women and their relation to life satisfaction. Dreaming.

[14] Fosse R, Stickgold R, Hobson JA (2001). The mind in REM sleep: reports of emotional experience. Sleep.

[15] Hall, C. S. & Van de Castle, R. L. *The content analysis of dreams. Central Psychology Series* (Appleton-Century-Crofts, 1966).

[16] Merritt JM, Stickgold R, Pace-Schott EF, Williams J, Hobson JA (1994). Emotion profiles in the dreams of men and women. Conscious. Cogn..

[17] Sikka P, Valli K, Virta T, Revonsuo A (2014). I know how you felt last night, or do I? Self- and external ratings of emotions in REM sleep dreams. Conscious. Cogn..

[18] Cartwright, R. D. *The twenty-four hour mind: the role of sleep and dreaming in our emotional lives*. (Oxford University Press, 2010).

[19] Hartmann, E. *The nature and functions of dreaming*. (Oxford University Press, 2010).

[20] Kramer, M. The selective mood regulatory function of dreaming: an update and revision. In *The functions of dreaming*. (eds Moffitt, A., Kramer, M. & Hoffmann, R.) 139–195 (State University of New York Press, 1993).

[21] Cook CAL, Caplan RD, Wolowitz H (1990). Nonwaking responses to waking stressors: dreams and nightmares. J. Appl. Soc. Psychol..

[22] De Koninck J, Koulack D (1975). Dream content and adaptation to a stressful situation. J. Abnorm. Psychol..

[23] Delorme MA, Lortie-Lussier M, De Koninck J (2002). Stress and coping in the waking and dreaming states during an examination period. Dreaming.

[24] Roberts J, Lennings CJ, Heard R (2009). Nightmares, life stress, and anxiety: an examination of tension reduction. Dreaming.

[25] Blagrove, M. & Pace-Schott, E. F. Trait and neurobiological correlates of individual differences in dream recall and dream content. In *International Review of Neurobiology: Dreams and Dreaming*. (eds Clow, A. & McNamara, P.) **92**, 155–180 (Elsevier, 2010). 10.1016/S0074-7742(10)92008-4 20870067

[26] Hartmann E, Rosen R, Rand W (1998). Personality and dreaming: boundary structure and dream content. Dreaming.

[27] Simor P, Köteles F, Sándor P, Petke Z, Bódizs R (2011). Mindfulness and dream quality: the inverse relationship between mindfulness and negative dream affect. Scand. J. Psychol..

[28] Duval M, McDuff P, Zadra A (2013). Nightmare frequency, nightmare distress, and psychopathology in female victims of childhood maltreatment. J. Nerv. Ment. Dis..

[29] Esposito K, Benitez A, Barza L, Mellman TA (1999). Evaluation of dream content in combat-related PTSD. J. Trauma. Stress.

[30] Helminen E, Punamäki R-L (2008). Contextualized emotional images in children’s dreams: psychological adjustment in conditions of military trauma. Int. J. Behav. Dev..

[31] Valli K (2005). The threat simulation theory of the evolutionary function of dreaming: evidence from dreams of traumatized children. Conscious. Cogn..

[32] Blagrove M, Farmer L, Williams E (2004). The relationship of nightmare frequency and nightmare distress to well-being. J. Sleep Res..

[33] Zadra A, Donderi DC (2000). Nightmares and bad dreams: their prevalence and relationship to well-being. J. Abnorm. Psychol..

[34] Levin R, Nielsen TA (2007). Disturbed dreaming, posttraumatic stress disorder, and affect distress: a review and neurocognitive model. Psychol. Bull..

[35] Levin R, Nielsen TA (2009). Nightmares, bad dreams and emotion dysregulation: a review and new neurocognitive model of dreaming. Curr. Dir. Psychol. Sci..

[36] Sandman N (2017). Nightmares as predictors of suicide: an extension study including war veterans. Sci. Rep..

[37] Schredl M, Engelhardt H (2001). Dreaming and psychopathology: dream recall and dream content of psychiatric inpatients. Sleep Hypn..

[38] Soffer-Dudek N (2017). Arousal in nocturnal consciousness: how dream- and sleep-experiences may inform us of poor sleep quality, stress, and psychopathology. Front. Psychol..

[39] Nielsen TA (2017). The stress acceleration hypothesis of nightmares. Front. Neurol..

[40] Hartmann, E. *Boundaries in the mind: a new psychology of personality*. (Basic Books, 1991).

[41] Sylvers P, Lilienfeld SO, LaPrairie JL (2011). Differences between trait fear and trait anxiety: implications for psychopathology. Clin. Psychol. Rev..

[42] Blagrove M, Fisher S (2009). Trait-state interactions in the etiology of nightmares. Dreaming.

[43] Schredl M, Schäfer G, Hofmann F, Jacob S (1999). Dream content and personality: thick vs. thin boundaries. Dreaming.

[44] Sándor, P., Horváth, K., Bódizs, R. & Konkolÿ Thege, B. Attachment and dream emotions: The mediating role of trait anxiety and depression. *Curr. Psychol*. 1–10, 10.1007/s12144-018-9890-y (2018).

[45] Nielsen TA (2000). Development of disturbing dreams during adolescence and their relation to anxiety symptoms. Sleep.

[46] Levin R, Fireman G, Spendlove S, Pope A (2011). The relative contribution of affect load and affect distress as predictors of disturbed dreaming. Behav. Sleep Med..

[47] Schredl M (2003). Effects of state and trait factors on nightmare frequency. Eur. Arch. Psychiatry Clin. Neurosci..

[48] Soffer-Dudek N, Shahar G (2011). Daily stress interacts with trait dissociation to predict sleep-related experiences in young adults. J. Abnorm. Psychol..

[49] Cellucci AJ, Lawrence PS (1978). Individual differences in self-reported sleep variable correlations among nightmare sufferers. J. Clin. Psychol..

[50] Malinowski JE, Horton CL (2014). Evidence for the preferential incorporation of emotional waking-life experiences into dreams. Dreaming.

[51] Watson D (2001). Dissociations of the night: individual differences in sleep-related experiences and their relation to dissociation and schizotypy. J. Abnorm. Psychol..

[52] Nielsen TA, Kuiken D, Alain G, Stenstrom P, Powell RA (2004). Immediate and delayed incorporations of events into dreams: Further replication and implications for dream function. J. Sleep Res..

[53] Eichenlaub J-B (2019). The nature of delayed dream incorporation (‘dream-lag effect’): personally significant events persist, but not major daily activities or concerns. J. Sleep Res..

[54] Agargun MY (2003). Nightmares and dissociative experiences: the key role of childhood traumatic events. Psychiatry Clin. Neurosci..

[55] Wittmann L, Schredl M, Kramer M (2007). Dreaming in posttraumatic stress disorder: a critical review of phenomenology, psychophysiology and treatment. Psychother. Psychosom..

[56] Duval M, Zadra A (2010). Frequency and content of dreams associated with trauma. Sleep Med. Clin..

[57] Aumann C, Lahl O, Pietrowsky R (2012). Relationship between dream structure, boundary structure and the big five personality dimensions. Dreaming.

[58] Zborowski M, McNamara P, Hartmann E, Murphy M, Mattle L (1998). Boundary structure related to sleep measures and to dream content. Sleep.

[59] Hartmann E, Russ D, Oldfield M, Sivan I, Cooper S (1987). Who has nightmares?. Arch. Gen. Psychiatry.

[60] Wood JM, Bootzin RR (1990). The prevalence of nightmares and their independence from anxiety. J. Abnorm. Psychol..

[61] Robert G, Zadra A (2008). Measuring nightmare and bad dream frequency: impact of retrospective and prospective instruments. J. Sleep Res..

[62] Beaulieu-Prévost D, Zadra A (2007). Absorption, psychological boundaries and attitude towards dreams as correlates of dream recall: two decades of research seen through a meta-analysis. J. Sleep Res..

[63] Wood JM, Bootzin RR, Rosenhan D, Nolen-Hoeksema S, Jourden F (1992). Effects of the 1989 San Francisco earthquake on frequency and content of nightmares. J. Abnorm. Psychol..

[64] Soffer-Dudek N, Shahar G (2010). Effect of exposure to terrorism on sleep-related experiences in Israeli young adults. Psychiatry Interpers. Biol. Process..

[65] Koulack D, Prevost F, De Koninck J (1985). Sleep, dreaming, and adaptation to a stressful intellectual activity. Sleep.

[66] Cartwright RD, Lloyd S, Knight S, Trenholme I (1984). Broken dreams: a study of the effects of divorce and depression on dream content. Psychiatry.

[67] Revonsuo, A., Tuominen, J. & Valli, K. Avatars in the machine: dreaming as a simulation of social reality. In *Open Mind: Philosophy and the Mind Sciences in the 21st Century* (eds Metzinger, T. & Windt, J. M.) **2**, 1295–1322 (MIND Group, 2016).

[68] Takahashi T (2005). Anxiety, reactivity, and social stress-induced cortisol elevation in humans. Neuroendocrinol. Lett..

[69] Tuominen J, Stenberg T, Revonsuo A, Valli K (2019). Social contents in dreams: an empirical test of the Social Simulation Theory. Conscious. Cogn..

[70] van Eck MM, Berkhof H, Nicolson NA, Sulon J (1996). The effects of perceived stress, traits, mood states, and stressful daily events on salivary cortisol. Psychosom. Med..

[71] Hirvikoski T, Lindholm T, Nordenström A, Nordström AL, Lajic S (2009). High self-perceived stress and many stressors, but normal diurnal cortisol rhythm, in adults with ADHD (attention-deficit/hyperactivity disorder). Horm. Behav..

[72] Nagy T (2015). Frequent nightmares are associated with blunted cortisol awakening response in women. Physiol. Behav..

[73] Payne, J. D. Memory consolidation, the diurnal rhythm of cortisol, and the nature of dreams: a new hypothesis. In *International Review of Neurobiology: Dreams and Dreaming* (eds Clow, A. & McNamara, P.) 92, 101–134 (Elsevier, 2010). 10.1016/S0074-7742(10)92006-0 20870065

[74] Carr, M. & Solomonova, E. Dream recall and content in different stages of sleep and time-of-night effect. In *Dreams: Understanding Biology, Psychology, and Culture* (eds Valli, K., Hoss, R. J. & Gongloff, R. P.) 188–194 (Greenwood Publishing Group, 2019).

[75] Trinder J, Kramer M (1971). Dream recall. Am. J. Psychiatry.

[76] Foulkes D, Sullivan B, Kerr NH, Brown L (1988). Appropriateness of dream feelings to dreamed situations. Cogn. Emot..

[77] Nielsen TA, Deslauriers D, Baylor GW (1991). Emotions in dream and waking event reports. Dreaming.

[78] Schredl M, Doll E (1998). Emotions in diary dreams. Conscious. Cogn..

[79] Sikka P, Feilhauer D, Valli K, Revonsuo A (2017). How you measure is what you get: differences in self- and external ratings of emotional experiences in home dreams. Am. J. Psychol..

[80] Kunzendorf RG, Hartmann E, Cohen R, Cutler J (1997). Bizarreness of the dreams and daydreams reported by individuals with thin and thick boundaries. Dreaming.

[81] Dowlatshahi Milad, Saghaiannejad Seyed Morteza, Ahn Jin-Woo, Moallem Mehdi (2014). Minimization of Torque-Ripple in Switched Reluctance Motors Over Wide Speed Range. Journal of Electrical Engineering and Technology.

[82] Gauthier J, Bouchard S (1993). Adaptation canadienne-française de la forme revisée du State-Trait Anxiety Inventory de Spielberger. Rev. Can. des Sci. du Comport..

[83] Hébert, M., Cyr, M. & Zuk, S. *Traduction et adaptation française du Early Trauma Inventory Self-Report - Short Form (ETISR-SF, 2007) de Bremner, Bolus et Mayer* (2008).

[84] Bremner JD, Bolus R, Mayer EA (2007). Psychometric properties of the Early Trauma Inventory-Self Report. J. Nerv. Ment. Dis..

[85] Prins A (2004). The primary care PTSD screen (PC–PTSD): development and operating characteristics. Prim. Care Psychiatry.

[86] Brantley PJ, Waggoner CD, Jones GN, Rappaport NB (1987). A daily stress inventory: development, reliability, and validity. J. Behav. Med..

[87] Woltman H, Feldstain A, MacKay C, Rocchi M (2012). An introduction to hierarchical linear modeling. Tutor. Quant. Methods Psychol..

[88] Hays William L. (1983). Review of Using Multivariate Statistics. Contemporary Psychology: A Journal of Reviews.

