# Supplementary material for: Predicting the affective tone of everyday dreams: A prospective study of state and trait variables
Source: Sci Rep. 2019 Oct 14;9:14780. doi: 10.1038/s41598-019-50859-w (PMC6791936; doi:10.1038/s41598-019-50859-w)
Supplement: Supplementary file 1 — Supplementary Tables [file 41598_2019_50859_MOESM1_ESM.pdf]

Predicting the affective tone of everyday dreams:
A prospective study of state and trait variables

Eugénie Samson-Daoust, Sarah-Hélène Julien, Dominic Beaulieu-Prévost & Antonio Zadra

Supplementary Tables

Supplementary Tables

	B	*SE*	*Wald* χ^2	*p*	95% Wald CI	
					LL	*UL*
Intercept	1.226	0.095	167.190	<0.001***	1.040	1.412
Between-subject predictors (Level-2)						
Sex	0.038	0.092	0.173	0.677	-0.142	0.219
Age	-0.003	0.004	0.851	0.356	-0.010	0.004
**Trait anxiety (STAI-T)**	**0.010**	**0.005**	**4.655**	**0.031***	**0.001**	**0.019**
Boundary thinness (BQ18)	0.004	0.005	0.557	0.455	-0.006	0.014
Youth trauma (ETISR-SF)	0.008	0.024	0.126	0.723	-0.038	0.054
PTSD symptoms (PC-PTSD)	-0.024	0.040	0.343	0.558	-0.102	0.055
Within-subject predictors (Level-1)						
Dream recall frequency (DRF)	0.011	0.038	0.086	0.770	-0.064	0.086
Dream valence	0.005	0.009	0.298	0.585	-0.013	0.023

Table S1. Generalized Estimating Equation Model with Poisson Distribution for Maximum Perceived Stress Level. *Note.* Age, trait anxiety and boundary thinness were grand mean centered (level-2), while dream valence was participant mean centered (level-1). *SE* = standard error. Based on 1,328 observations in 128 participants. **p* < 0.05. ***p* < 0.01. ****p* < 0.001.

	B	*SE*	*Wald* $\chi2$	*p*	95% Wald CI	
					LL	*UL*
Intercept	0.560	0.206	7.356	0.007**	0.155	0.964
Between-subject predictors (Level-2)						
Sex	-0.059	0.181	0.104	0.747	-0.414	0.297
Age	0.002	0.006	0.141	0.708	-0.010	0.015
**Trait anxiety (STAI-T)**	**0.024**	**0.009**	**7.378**	**0.007****	**0.007**	**0.040**
Boundary thinness (BQ18)	0.003	0.010	0.072	0.789	-0.017	0.022
Youth trauma (ETISR-SF)	-0.045	0.047	0.929	0.335	-0.136	0.046
PTSD symptoms (PC-PTSD)	0.039	0.072	0.289	0.591	-0.102	0.179
Within-subject predictors (Level-1)						
Dream recall frequency (DRF)	-0.018	0.075	0.059	0.809	-0.165	0.128
Dream valence	-0.004	0.013	0.080	0.778	-0.028	0.021

Table S2. Generalized Estimating Equation Model with Poisson Distribution for Bedtime Perceived Stress Level. *Note.* Age, trait anxiety and boundary thinness were grand mean centered (level-2), while dream valence was participant mean centered (level-1). *SE* = standard error. Based on 1,329 observations in 128 participants. **p* < 0.05. ***p* < 0.01. ****p* < 0.001.
